# Azolla pinnata as a phytoremediator: improves germination, growth and yield of maize irrigated with Ni-polluted water

**DOI:** 10.1038/s41598-024-72651-1

**Published:** 2024-09-27

**Authors:** Ibrahim Zeid, Essra Khaled Ghaly, Zeinab Ashour Shedeed

**Affiliations:** Botany and Microbiology Department, Faculty of Science, Helwan University, Cairo, Egypt

**Keywords:** Heavy metals, Oxidative stress, Photosynthesis, Stomatal conductance, Biochemistry, Ecology, Physiology, Plant sciences, Environmental sciences, Natural hazards

## Abstract

The removal of pollutants from the environment has become a global demand. The current study aimed to relieve the Ni toxicity effect on the germination, growth, and grain yield of maize by using *Azolla pinnata* as a phytoremediator. *Azolla-*treated and untreated nickel solutions [0 (control), 24, 70, 140 and 190 ppm] were applied for germination and pot experiments. Electron microscope examination cleared the Ni accumulation in *Azolla’s* cell vacuole and its adsorption on the cell wall. The inhibition of the hydrolytic enzyme activity reduces maize germination; maximal inhibition was 57.1% at 190 ppm of Ni compared to the control (100%). During vegetative growth, Ni stimulated the generation of H_2_O_2_ (0.387 mM g^−1^ F Wt at 190 ppm of Ni), which induced maximal lipid peroxidation (3.913 µMDA g^−1^ F Wt) and ion leakage (74.456%) compared to control. Chlorophyll content and carbon fixation also showed significant reductions at all Ni concentrations; at 190 ppm, they showed maximum reductions of 56.2 and 63%, respectively. However, detoxification enzymes’ activity such as catalase and antioxidant substances (phenolics) increased. The highest concentration of Ni (190 ppm) had the most effect on constraining yield, reaching zero for the weight of 100 grains at 190 ppm of Ni. *Azolla*-treated Ni solutions amended all determinant parameters, indicating a high percentage of changes in hydrolytic enzyme activity (125.2%) during germination, chlorophyll content (77.6%) and photosynthetic rate (120.1%). Growth measurements, carbon fixation, and yield components showed a positive association. Thus, we recommended using *Azolla* as a cost-effective and eco-friendly strategy to recover Ni-polluted water.

**Supplementary Information:**

The online version contains supplementary material available at 10.1038/s41598-024-72651-1.

## Introduction

Heavy metal build-up in soil has grown significantly because of natural processes as well as human (industrial) activity^1^. The presence of various pollutants such as organic or and inorganic elements in landfills have a global interest. Such toxins and heavy metalloids can represent a main danger to human health as well as eco-toxic effects on different ecosystems (terrestrial and aquatic)^2^. Heavy metals, such as arsenic (As), cadmium (Cd), chromium (Cr), mercury (Hg), lead (Pb), copper (Cu), zinc (Zn), and nickel (Ni), are prevalent contaminants in the soil environment. This form of pollution is physiologically hazardous, widespread, and long-lasting in the soil environment^3^.

Nickel is considered an essential element for the growth of plants, and its deficiency has been observed in some perennial species^4^. Primary resources of Ni may be natural processes such as erosion of rock, weathering, and volcanic explosion) or human-causes such as industrial discharges, mine actions, electroplating and municipal sewage sludge that caused contamination in ecosystems^5–7^. Increasing Ni levels above the permitted levels in water (0.02 mg l^−1^) and soil (35 mg kg^−1^) causes harmfulness to all living organisms^8,9^.

Nickel is a necessary micronutrient in plants because it is a component of the active site of the urease enzyme which hydrolyzes urea in plant tissues^10^. Excess concentrations of Ni in plants cause chlorosis and necrosis, as with other heavy metals, disrupt Fe uptake and metabolism^11^. Nickel’s toxic effects on plants include changes in the germination process, total dry matter production and yield, all of which have a negative impact on the plant. The activity of hydrolytic enzymes was enhanced at lower Ni concentrations and decreased at higher ones^12^. Lethal levels of Ni damage plants by disturbing a range of physiological functions (including enzyme activity), root progress, photosynthesis, and element uptake^13^.

Nickel has a substantial impact on plant metabolic processes because of its capacity to form reactive oxygen species that can induce oxidative stress^14^. When heavy metals are present in plants, they quickly assemble into reactive oxygen species (ROS), resulting in oxidative stress^15^. Plants are bestowed with a variety of enzymatic and non-enzymatic antioxidants that aggravate their ability to scavenge excess ROS and, in that way, play a critical role in ROS homeostasis. Antioxidant enzymes in plants include superoxide dismutase (SOD), ascorbate peroxidase (APX), catalase (CAT) and glutathione peroxidase (GPX)^16^.

Moreover, Ni toxicity affects photosynthesis and gas exchange processes in many ways, resulting in an overall inhibition of photosynthesis^12^. The drop in chlorophyll concentration caused by the Ni treatment may have reduced the chloroplast’s capacity to absorb light, indirectly impairing photosynthesis. Chlorophyll fluorescence characteristics are a good measure of the level of abiotic stress, and photosystem II (PS II) is particularly susceptible to damage from metal stress^17^. Li et al.^18^ recently demonstrated that *Elsholtzia argyi’s* decreased capacity for photosynthesis under heavy metal stress is correlated with decreased photochemical efficiency (PS), photochemical quenching (qP), electron transport rate (ETR), and non-photochemical quenching (NPQ).

Metal contamination of soil is an environmental risk and chemical treatments for heavy metal decontamination are generally very expensive and not applicable to agricultural lands. Therefore, many strategies are used to restore contaminated areas. Phytoremediation is a promising method based on the use of hyper-accumulator plant species that can tolerate toxic heavy metals in the environment^19^. Aquatic phytoremediation removes contaminants from water and restores damaged water bodies. Aquatic phytoremediation takes place by macrophytes (freshwater-adapted angiosperms, pteridophytes, and ferns) to remove and reduce pollutants in aquatic bodies^20^. Those macrophytes are able to accumulate or breakdown pollutants by rhizo/phyto-filtration, phyto-extraction, phyto-volatilization, and phyto-degradation.

Maize is one of the world’s most popular, oldest, and most potent cereal crops, used for food, fodder, and even medicine. More than 3500 applications for corn products have been proposed. Because of its nutritional value, it addresses health-related concerns. Maize is also high in vitamins A, B, and E, as well as a variety of minerals. It has decreased hypertension and helped to avoid neural-tube abnormalities in children. According to Lasat^21^ metal hyperaccumulators as maize offer several benefits, but they may be sluggish to develop and generate little biomass, making cleanup of polluted locations time-consuming. To overcome these obstacles, some scientists proposed the utilization of metal chelators to improve chemical phytoextraction. The strategy utilizes high-biomass crops that are chemically treated with chelating organic acids to boost their mobility in soil, causing them to absorb significant quantities of metals^22^. *Azolla* can be used as a natural low-cost effective chelator to extract metals from soil, reducing their availability in soil let maize uptake less metal.

The responses of maize when it faces more than one stress (double or triple) has been received more interest, such as drought plus heat or heavy metal exposure. To meet these issues in maize, transgenic approaches have already caused the production of viable stress resistant variants; natural variation and genetic engineering are used^23^. On the one hand, quantitative trait loci (QTL) linked with multiple-stress tolerance are being used via molecular breeding and genome-wide association studies (GWAS), which might be used in future breeding efforts for more robust maize varieties^23^.

The aim of this study was to use *Azolla pinnata* as an eco-friendly remediator to improve maize plant growth under Ni-induced stress. *Azolla pinnata* was used to relieve the inhibitory effects of Ni-polluted solutions on maize germination, vegetative growth, photosynthetic capacity and grain yield. It is inventive to use *Azolla* in Ni-contaminated soil that is cultivated with maize.

## Materials and methods

### Materials

Seeds of *Zea mays* were kindly obtained from Legumes Crops Department; Field Crops Research Institute; Agricultural Research Centre, Giza, Egypt. *Azolla Pinnata* was obtained from Water and Land Department; Field Crops Research Institute; Agricultural Research Centre, Giza, Egypt.

### Preparation of *Azolla*-treated Ni solutions

**Fig. 1:**
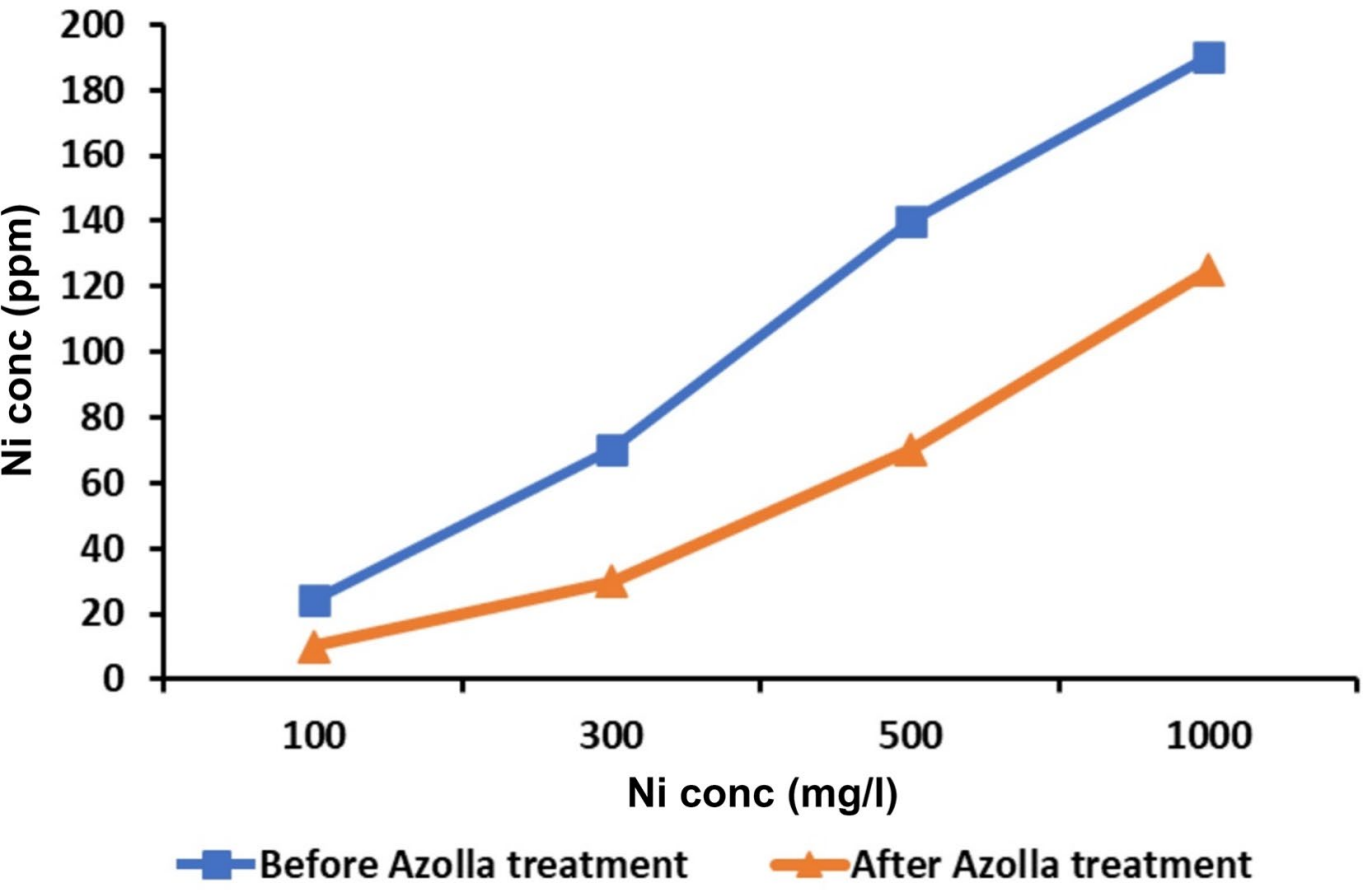
Concentration of Ni ion measured by (MPAES, Agilent, Santa Clara, CA 95051, United States) in a series of nickel chloride solutions (100, 300, 500 and 1000 mg/l) before and after *Azolla pinnata* treatment for 4 days.

To assess the toxicity of nickel chloride (NiCl_2_) on the germination of maize seeds, various concentrations of NiCl_**2**_ (0, 100, 300, 500, 1000 and 2000 ppm) were used (preliminary experiment). The lethal concentration was 2000 ppm NiCl_**2**_. Five conical flasks (250 ml capacity) with different Ni solutions and water (0, 100, 300, 500 and 1000 ppm) were inoculated with 10 g of fresh *Azolla* (10%, wt/v). After four days of incubation, Ni solutions were filtered through filter paper and the concentration of Ni ions for each original and *Azolla-*treated Ni solutions was determined by using a Microwave Plasma Atomic Emission Spectrometer (MPAES, Agilent, Santa Clara, CA 95051, United States). The concentrations of Ni ions were represented in Fig. 1 before and after *Azolla* treatment.

#### Laboratory experiment (germination stage)

Maize seeds were sterilized in 2.5% sodium hypochlorite solution (3 min). Then, seeds washed with distilled water. In petri dishes, seven seeds were distributed on filter paper. For each petri-dish, seven milliliters of different Ni solutions [0 (control), 24, 70, 140 and 190 ppm] or Ni solutions that were previously treated with *Azolla* were added. Once the radicle had grown to around 2 mm, the percentage of germination was recorded on the fourth day of germination. Germination parameters and biochemical analyses were determined on the seventh day of germination.

#### Pot experiment (vegetative growth and yield stage)

The experiment was carried out in the autumn (November, 2022) at the Botany and Microbiology Department, Faculty of Science, Helwan University. The sieved clay was mixed well with sieved clean sand. In wide-mouthed glazed pots of diameter 70 cm, 5 kg of clay-sand soil was added. The pots were divided into two groups; each group involved five treatments. The first group was represented by the original Ni solutions, including water (0 (control), 24, 70, 120, 190 ppm). The second group was represented by the *Azolla*-treated-Ni solutions, including water (0 (control), 24, 70, 120 and 190 ppm). Each treatment was represented in triple (*n* = 3). After 30 days of sowing, the plants were collected for growth metrics and biochemical analysis. After about 6 months, the grain yield was recorded.

### Biochemical analyses

#### Enzyme activity assay

##### Hydrolases

Double-distilled water was used to grind a known weight of germinated maize seeds into a paste, which was subsequently centrifuged for 20 min at 4 °C at 4000 rpm. The enzyme assay was conducted using the clear supernatant solution according to Zeid^24^. α-amylase (E.C. 3.2.1.1) and protease enzyme (E.C.3.4) activities were measured according to Bergmeyer^25^. For amylase, half milliliter of soluble starch in phosphate buffer (pH 7.0), 0.5 ml of double-distilled water, and 0.5 ml of enzyme extract made up the tested combination. One milliliter of the coloring reagent (1% dinitrosalisylic acid) was added to the mixture after a ten-minute incubation time at 25 °C and the mixture was then heated in a water bath for ten minutes before cooling in an ice bath. Double-distilled water was added to the mixture to make 10 ml, and a spectrophotometer (CE 1010) was used to detect the color density at 546 nm. For protease, 1 ml of casein (1%) in phosphate buffer (pH 7.5) and 1 ml of enzyme extract made up the assaying mixture. The reaction was stopped after an hour of incubation at 37 °C with the addition of 2 ml of 10% trichloroacetic acid, and the mixture was then centrifuged at 4000 rpm for 20 min at 4 °C. According to Lowry et al.^26^, the supernatant’s soluble peptide content was calculated. Three replicates of each determination were made (*n* = 3).

##### Antioxidant enzymes extraction

The supernatant that was used to evaluate enzyme activity was prepared according to Castillo et al.^27^. In a prechilled pestle and mortar, 0.5 g of fresh leaf material was homogenized with 10 ml of ice-cold phosphate buffer (Na/KP), pH 6.8. The homogenate was centrifuged for 10 min at 6000 rpm at 4 °C after being filtered through cheesecloth. Catalase (EC 1.11.1.6) activity was assessed according to the Góth^28^. The assaying mixture contained 0.2 ml of the enzyme extract and 1 ml of H_2_O_2_ (65 mM H_2_O_2_ in Na/KP, pH 7.4). By adding 1 ml of ammonium molybedate (4 g/l), the reaction was stopped after an incubation period of 4 min at 25 °C. Using a UV spectrophotometer (6405 UV/Vis), the generated color’s absorbance was determined at 405 nm.

For peroxidase activity, peroxidase (EC 1.11.1.7) activity was assessed according to the Yamane et al.^29^ method. The assaying mixture contains 0.2 ml 30% H_2_O_2_, 0.5 ml 8 mM guaicol, and 2.2 ml 10 mM potassium phosphate buffer. After pouring the assaying mixture into a clean quartz cuvette, the reaction was started by adding 0.1 ml of enzyme extract and mixing right away. The cuvette was put inside the UV spectrophotometer (6405 UV/Vis) to track absorbance changes for up to three minutes at 470 nm.

#### Chemical analysis

##### Photosynthetic pigments content and measurement of gas exchange

The Metzener et al.^30^ approach, as modified by Lichtenthaler^31^, was used to estimate the pigment content. Fresh leaves of a given weight were homogenized in 85% acetone. After 20 min of centrifugation at 4000 rpm and 4 °C, the pigment-containing supernatant was diluted to a specific volume with 85% acetone. Using a spectrophotometer and 85% acetone as a blank, the supernatant was measured at two different wave lengths on the spectrophotometer (CE 1010): 645 nm and 664 nm. Three replicates of each determination were made (*n* = 3). The pigments were measured in mg g^−1^ Dwt of tissue. According to the following formulae, the concentrations of chlorophyll a and chlorophyll b were determined:

$${\text{Chlorophyll}}\,{\text{a}}\,=\,10.3{\text{ E}}664--0.918{\text{ E}}645,{\text{ Chlorophyll b}}\,=\,19.7{\text{ E}}645--3.87{\text{ E}}664$$

The gaseous exchange of each control and treated maize plant was measured by a portable photosynthesis system (LCpro-SD, ADC BioScientific, Hoddesdon, UK) with a standard 2 × 3 cm^2^ leaf chamber. The pots were well watered a day before measurements. The measurements were carried out on fully expanded leaves (3 leaves). The measurements were carried out in ambient light at a leaf temperature of 23 °C. The photosynthetic parameters were measured after 30 days of cultivation. The measured attributes include the photosynthetic rate (A), stomatal conductance (Gs), internal CO_2_ (Ci) and evaporation rate (E).

##### Total soluble sugars

Fresh leaves (0.1 g) were crushed with 5 ml of ethanol and centrifuged at 4 °C at 4000 rpm for 10 min. Then, the total soluble sugars were determined using the anthrone technique by Umbriet et al.^32^. Three replicates of each determination were made (*n* = 3).

##### Total soluble proteins

The Lowry et al.^26^ method used for total soluble proteins determination. A sample of the extract was treated with one millilitre of freshly mixed (1:1 v/v) solutions of 2% sodium carbonate in 4% sodium hydroxide and 0.5% copper sulphate in 1% sodium tartarate. The mixture was allowed to sit at room temperature for 10 min before adding 0.1 ml of Folin reagent. After 30 min, the optical density at 700 nm was measured using a spectrophotometer (CE 1010). Three replicates of each determination were made (*n* = 3).

##### Proline

Proline was extracted and estimated via the Bates et al.^33^ method by using an acid-ninhydrin reagent in glacial acetic acid.

##### Lipid peroxidation (Malondialdehyde content, MDA)

Monodehydroascorbate (MDA) was measured by the thiobarbituric acid (TBA) reaction to determine the level of lipid peroxidation as described by Doblinski et al.^34^. Fresh plant material (0.5 g) was homogenized in 10 ml of 5% trichloroacetic acid (TCA). Then, the mixture was centrifuged at 15,000*g* for 10 min. Four milliliters of thiobarbituric acid (0.5%) were added to 2 ml of the supernatant. At 95 °C, the previous mixture was heated for 30 min. Then, the mixture was cooled in an ice bath and centrifuged at 10,000*g* MDA equivalents were calculated using the Heath and Packer^35^ equation:

$${\text{MDA equivalents }}\left( {{\text{nmol c}}{{\text{m}}^{ - 1}}} \right)\,=\,1000{\text{ }}\left[ {\left( {Abs{\text{ }}532 - {\text{ }}Abs{\text{ }}600\,{\text{nm}}} \right)/155} \right].$$

##### Permeability plasma membranes (total electrolyte leakage)

The electrolytic conductivity of fresh leaves segments (10), 2.5 g, in 25 ml of deionized water (for 5 h) and after boiling (30 min) was measured by a conductivity meter. Three replicates of each determination were made (*n* = 3). According to Zwiazek and Blake’s^36^ explanation, the relative permeability of the root.

Electrolytic conductivity of solution at 5 h before heating × 100.

Electrolytic conductivity of solution after heating.

##### Hydrogen peroxide content

According to Velikova et al.^37^ hydrogen peroxide content was determined. Fresh plant material (0.5 g) was homogenized in an ice bath with 5 ml of 0.1% trichloroacetic acid. The homogenate was centrifuged for 15 min at 12,000 rpm. The supernatant was used to determine the H_2_O_2_ content by using a buffered potassium iodide (KI) reagent on a spectrophotometer at 390 nm. Three replicates of each determination were made (*n*=3).

##### Phenolic and flavonoid compounds

Phenolics were determined according to Savitree et al.^38^ by using a Folin-Ciocalteu reagent. The content of the total flavonoids was estimated by the ZhiShen et al.^39^ method, by mixing the extract with NaNO_2_, a 10% AlCl_3_ solution, and a 1% NaOH solution. Three replicates of each determination were made (*n* = 3).

##### Determination of Nickel content

A known weight of plant material was ashed and digested with nitric acid (HNO_3_)^40^, for subsequent determination by Microwave Plasma Atomic Emission Spectrometer (MPAES, Agilent, Santa Clara, CA 95051, United States). The translocation factor (TF) was used to estimate the translocation of Ni from the root to the leaves and seeds of maize^41^ as follows: TF_leaf_ = C _plant leaves_/C _plant roots_.

### Electron microscopy examination of *Azolla pinnata* cells

Stained sections were examined using a JEOL—JEM 1010 transmission electron microscope at 70 kV at Al-Azhar University’s Regional Centre for Mycology and Biotechnology (RCMB)^42^. The samples were fixed in 3% glutaraldehyde, rinsed in phosphate buffer, and post-fixed in potassium permanganate solution (0.1 g in 10 ml distalled water) for 5 min at room temperature. The samples were dehydrated in an ethanol series ranging from 10 to 90% for 15 min in each alcohol dilution and finally with absolute ethanol for 30 min. Samples were infiltrated with epoxy resin and acetone through a graded series until they were finally pure resin. Sample capsules were placed in an oven at 40 °C for 2 days to be sure that all the acetone had evaporated. Ultrathin Sect. (70 nm) were collected on copper grids. Sections were then double stained in uranyl acetate, followed by lead citrate.

### Statistical analysis

A one-way ANOVA (Tukey, post-hoc) was applied to assess the difference among each group’s means. Variations in the germination, growth parameters, enzymes activity and metabolite concentrations, Ni concentration and yield components under different Ni stress levels with and without *Azolla* were carried out by one-way analysis of variance (ANOVA I), and the mean values of three replicates were compared by a Tukey multiple comparison test at a 5% probability level. When the differences were significant, a post-hoc test (Tukey test at *P* < 0.05) was applied using SPSS (SPSS base 15.0 user’s guide, Chicago: SPSS 523 Inc.).

## Results

**Fig. 2:**
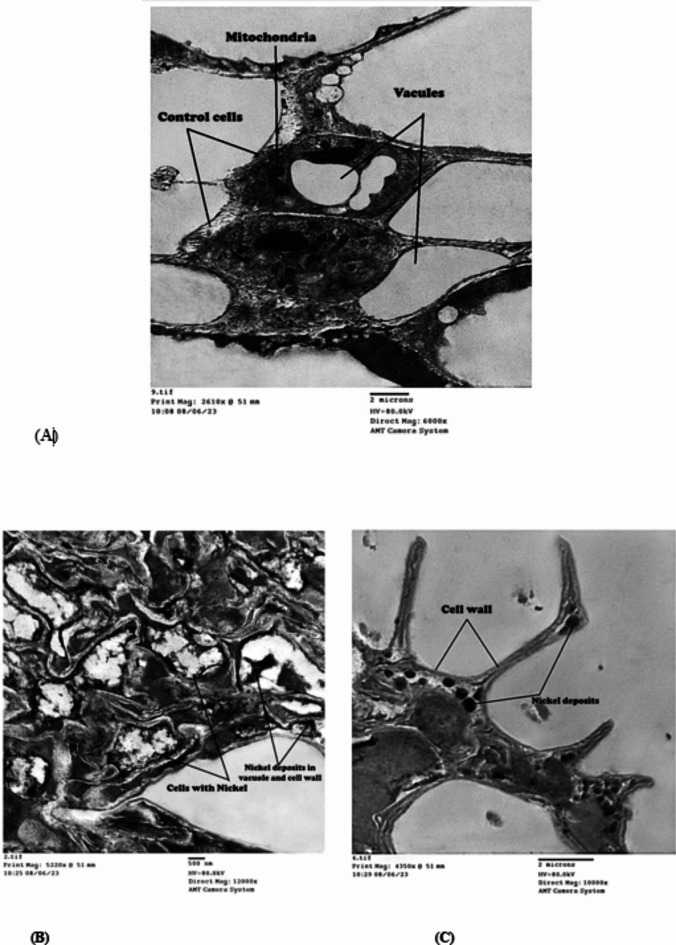
The desposition of Ni in *Azolla pinnata* vacuole and cell wall after incubation in Ni solution (190 pp ppm) for four days.(**A**) represents the control cell without Ni desposition; (**B**) and (**C**) respresent the Ni desposition in vacuole and on cell wall.

**Fig. 3:**
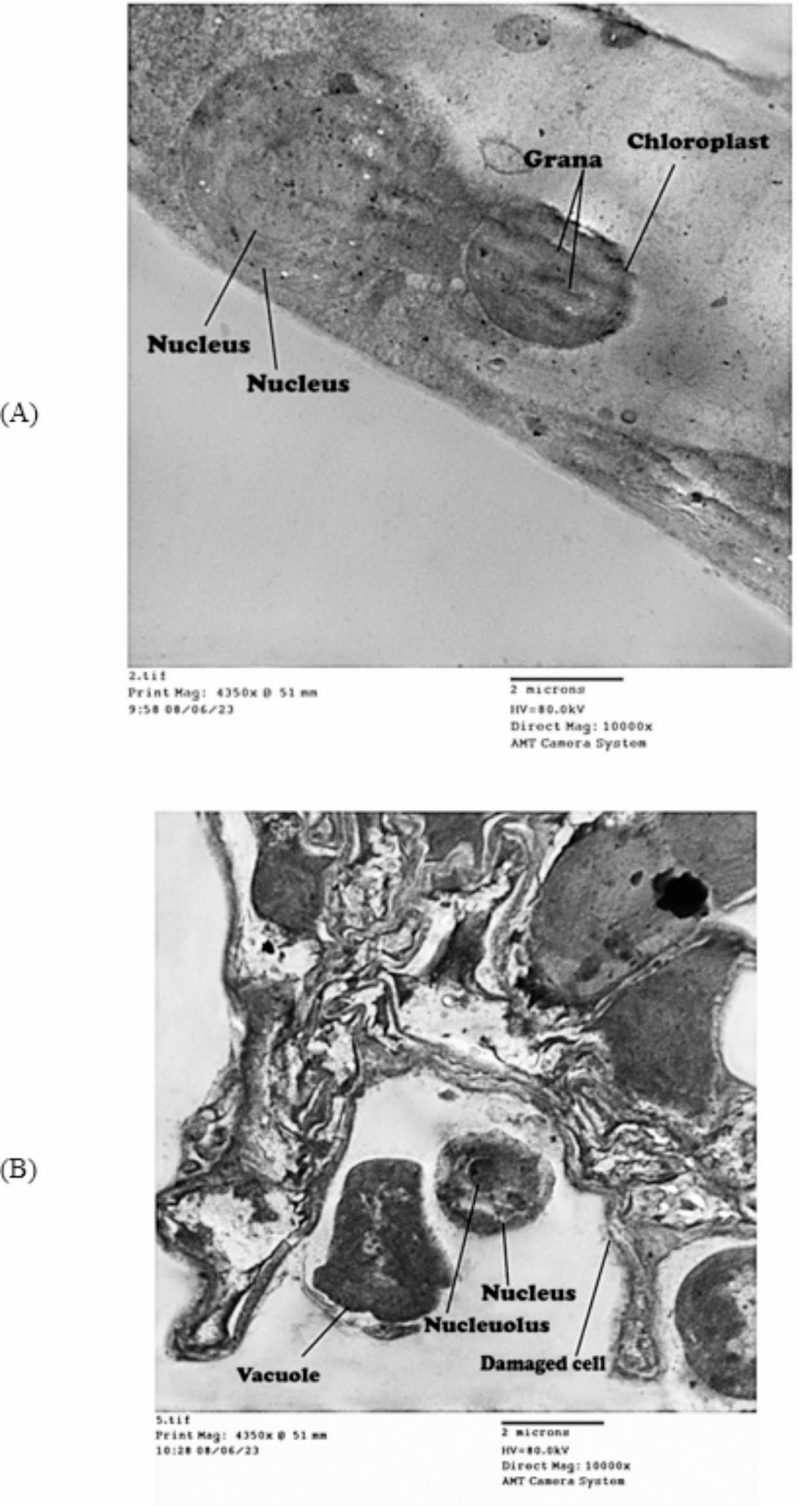
(**A**) represents the normal *Azolla pinnata* cell with nucleus and chloroplast; (**B**) represents the damaged cell of *Azolla pinnata* with Ni deposition in vacuole.

Nickel ion concentration was measured before and after *Azolla* treatment (Fig. 1); its concentration was 0, 24, 70, 140 and 190 ppm in the prepared NiCl_2_ solutions at 0, 100, 300, 500 and 1000 ppm concentrations before *Azolla* treatment. *Azolla* treatment reduced its concentration to become 0, 10, 30, 70 and 125, respectively. The removal efficiency percentage of Ni by *Azolla* was 58.3, 57.1, 50.0 and 34.2% for 24, 70, 140 and 190 ppm Ni-solutions, respectively. *Azolla* absorbed Ni ions from the external Ni polluted solutions and accumulated them in the vacuole, appearing as Ni deposits adsorbed on the *Azolla’s* cell wall (Fig. 2B,C) by transmission electron microscope (TEM) examination. The control cell appeared with no Ni deposits and a clear vacuole (Fig. 2A). The healthy *Azolla* cells appeared with normal chloroplasts and nuclei (Fig. 3A), while the *Azolla* incubated in Ni solutions (190 ppm) was damaged (Fig. 3B), appearing with an abnormal cell wall and disruption of organelles.

### The impact of untreated and *Azolla*-treated Ni-contaminated water on the germination of maize seeds

**Fig. 4:**
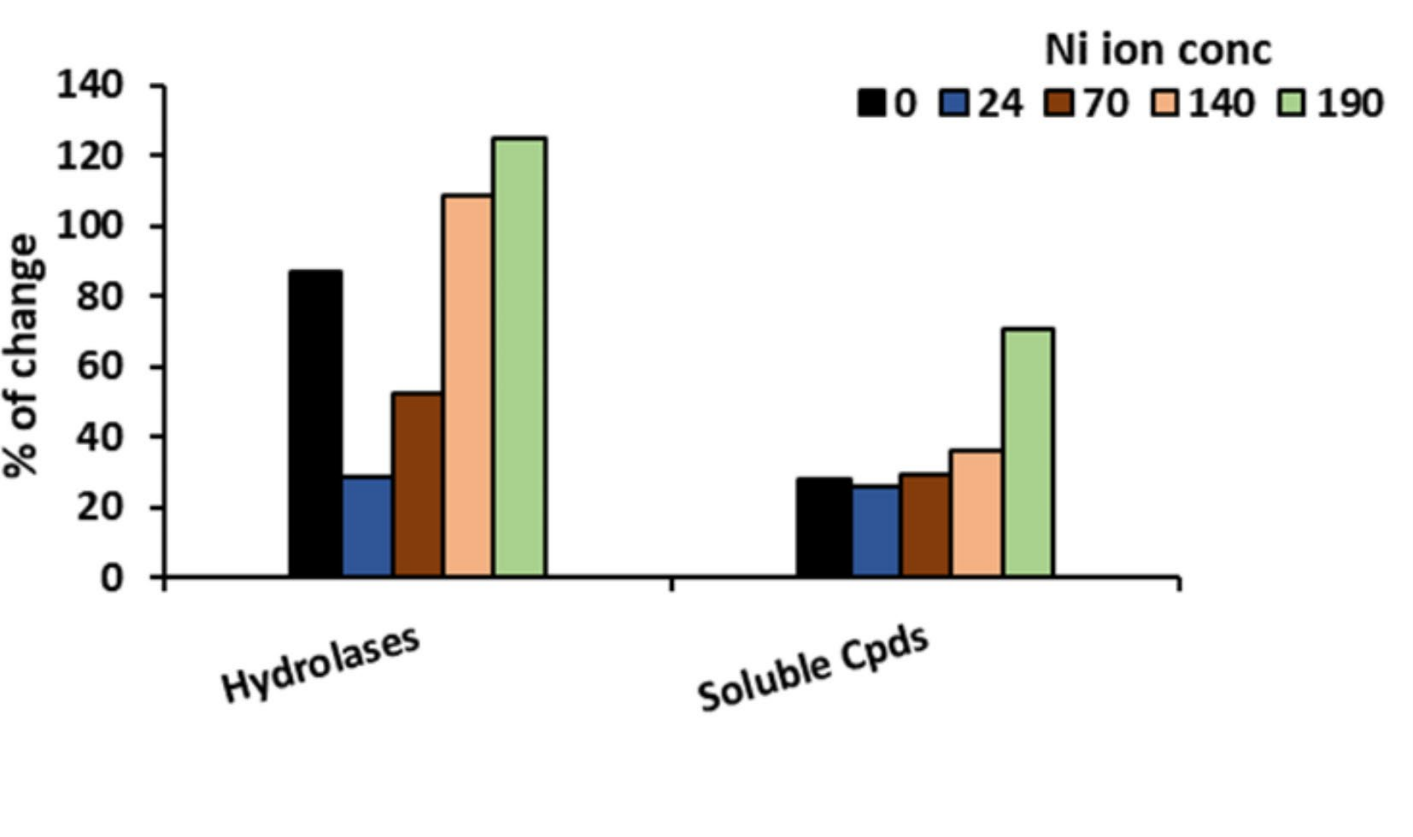
The percentage of change in hydrolases (α-amylase and protease) activity, total soluble compounds (total soluble sugars and proteins) content in response to *Azolla pinnata* treatment.

The current study found that increasing Ni concentration up to 190 ppm in the germination medium resulted in a progressive decrease in germination%, accompanied by a significant (at p < 0.05) reduction in hydrolytic enzymes activity, total soluble sugars and protein content (Table 1). Concerning the activity of the antioxidant enzymes, peroxidase (POX) and catalase (CAT) activities were increased at all Ni concentrations (Supplementary material S1), reaching their maximum activity at 190 ppm. Allowing the maize seeds to germinate in Ni-solutions previously treated with *Azolla* significantly decreased the inhibitory effect of Ni on the germination percentage, hydrolytic enzymes activity compared to un-treated ones (Table 1). The increment percentage in germination was 5, 23, 12 and 41% at 24, 70, 140 and 190 ppm Ni concentrations, respectively. The percentage of changes in germination parameters in response to *Azolla* treatment is represented in Fig. 4. The highest percentage of changes in the hydrolase’s activity and content of soluble sugars and proteins was at the highest concentration of Ni (190 ppm). The percentage of changes in germination parameters indicates the positive efficiency of *Azolla* in removing Ni from polluted solutions.

**Table 1:** Effect of different concentrations of Ni ion (0, 24, 70, 140, 190 ppm) before and after *Azolla piñnata* treatment on the germination %, activity of hydrolases (amylase and protease) [mg g^−1^ F wt min^−1^], total soluble sugars and proteins content [mg g^−1^ D wt] of maize seeds (7-day-old).

Ni ion conc (ppm)	Treatment	Germination	α-amylase	Protease	Total soluble sugars	Total soluble proteins
0	Before *Azolla* treatment	100.0 ± 0.00^a^	0.404 ± 0.004^b^	3.46 ± 1.12^bc^	31.22 ± 0.46^c^	11.53 ± 0.03^b^
24		95.20 ± 8.50^ab^	0.390 ± 0.001^c^	3.54 ± 0.09^bc^	30.58 ± 1.10^cd^	10.47 ± 0.27^de^
70		80.94 ± 8.20^cd^	0.206 ± 0.001^e^	2.34 ± 0.26^cde^	28.63 ± 0.12^de^	9.740 ± 0.14^ef^
140		76.06 ± 0.23^d^	0.187 ± 0.001^f^	1.51 ± 0.10^de^	18.55 ± 0.58^g^	9.040 ± 0.04^f^
190		57.10 ± 0.00^e^	0.094 ± 0.004^h^	1.26 ± 0.19^e^	11.86 ± 0.98^h^	7.660 ± 0.00^g^
0	After *Azolla* treatment	100.0 ± 0.00^a^	0.414 ± 0.001^a^	6.81 ± 0.50^a^	41.63 ± 0.37^a^	13.01 ± 0.08^a^
24		100.0 ± 0.00^a^	0.400 ± 0.001^b^	4.68 ± 1.08^b^	40.31 ± 0.31^ab^	11.27 ± 0.06^bc^
70		100.0 ± 0.00^a^	0.268 ± 0.005^d^	3.61 ± 0.12^bc^	38.94 ± 1.97^b^	10.57 ± 0.21^cd^
140		85.71 ± 0.34^bc^	0.206 ± 0.006^e^	3.34 ± 0.27^bc^	27.83 ± 0.75^e^	9.710 ± 0.40^f^
190		80.94 ± 7.90^cd^	0.150 ± 0.001^g^	2.90 ± 0.50^cd^	24.40 ± 0.00^f^	9.000 ± 0.61^f^

Means with the same letters are not significant according to Tukey test at 0.05. Each mean value followed by ± standard deviation.

### Growth parameters

All measured growth parameters (lengths, fresh and dry masses of maize roots and shoots) were obviously (p < 0.05) reduced with increasing Ni concentration. On the other side, *Azolla-*treated Ni-solutions amended the growth measurements of the plants (Table 2). Applying *Azolla* showed significant improvements for the shoot and root of maize compared to the untreated solutions, especially for fresh weights.

**Table 2:** Effect of different concentrations of Ni ion (0, 24, 70, 140, 190 ppm) before and after *Azolla piñnata* treatment on growth parameters of maize plants after 30 days from cultivation.

Ni ion conc (ppm)	Treatment	Length (cm)		Fresh Wt (g)		Dry Wt (g)	
		Shoot	Root	Shoot	Root	Shoot	Root
0	Before *Azolla* treatment	33.69 ± 0.58^b^	15.20 ± 0.40^b^	4.36 ± 0.28^a^	3.10 ± 0.167^d^	0.577 ± 0.012b	0.411 ± 0.0025^b^
24		31.58 ± 0.40^c^	14.63 ± 0.23^b^	3.60 ± 0.17^b^	2.84 ± 0.037e	0.522 ± 0.001^c^	0.371 ± 0.001^cd^
70		28.33 ± 0.20^e^	13.13 ± 0.15^cd^	3.37 ± 0.11^b^	2.66 ± 0.028ef	0.443 ± 0.002^e^	0.330 ± 0.001^cd^
140		27.07 ± 0.06^f^	12.00 ± 0.50^de^	3.10 ± 0.17^bc^	2.55 ± 0.050e	0.415 ± 0.005^f^	0.303 ± 0.005^f^
190		27.07 ± 0.06^f^	12.00 ± 0.50^de^	3.10 ± 0.17^bc^	2.55 ± 0.050e	0.415 ± 0.005^f^	0.255 ± 0.005^g^
0	After *Azolla* treatment	35.46 ± 0.17a	16.60 ± 0.17a	4.87 ± 0.10^a^	3.86 ± 0.032a	0.612 ± 0.002^a^	0.503 ± 0.003^a^
24		34.25 ± 0.05b	15.37 ± 0.13b	4.62 ± 0.28^a^	3.69 ± 0.040ab	0.593 ± 0.002^b^	0.383 ± 0.010^c^
70		30.33 ± 0.14d	14.30 ± 0.26bc	4.58 ± 0.10^b^	3.53 ± 0.026b	0.485 ± 0.005^d^	0.362 ± 0.002^e^
140		29.52 ± 0.13d	12.43 ± 0.51d	3.79 ± 0.04^b^	3.32 ± 0.020c	0.468 ± 0.007^d^	0.325 ± 0.005^e^
190		28.00 ± 0.50ef	11.00 ± 0.86e	3.42 ± 0.13^b^	2.69 ± 0.010ef	0.418 ± 0.007^f^	0.303 ± 0.005^f^

Means with the same letters are not significant according to Tukey test at 0.05. Each mean value followed by ± standard deviation.

### Photosynthetic pigments and efficiency

**Fig. 5:**
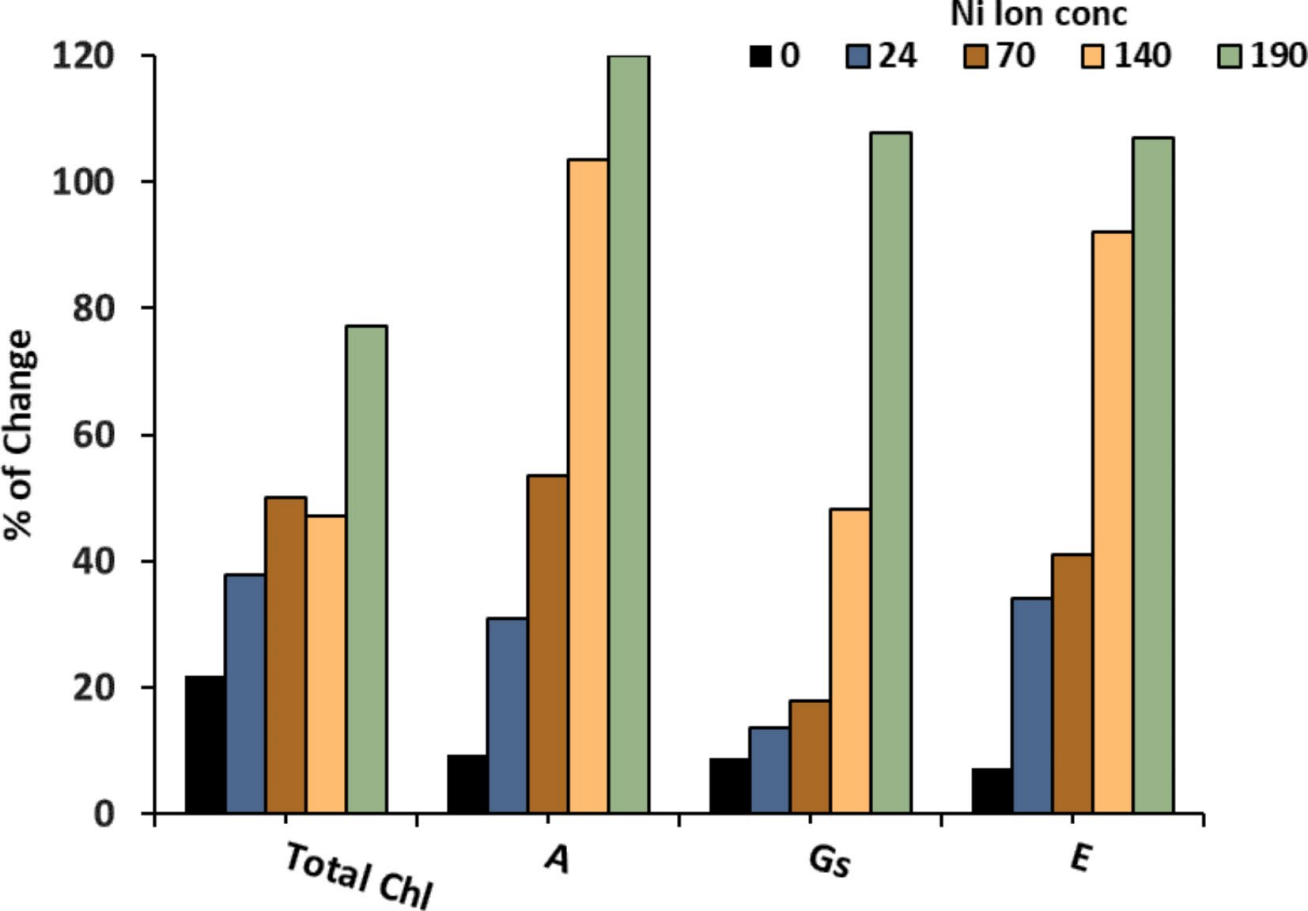
The percentage of change in total chl and photosynthetic capacity traits A (photosynthetic rate), Gs (stomatal conductance), E (transpiration rate) in response to *Azolla pinnata* treatment (the percentage difference between untreated and *Azolla*-treated Ni solutions effect on photosynthetic activity parameters).

Application of different Ni-solutions negatively affected the chlorophyll a and b content (Table 3) and the total Chl content showed the same trend for each individual Chl. Additionally, increasing Ni concentration in the growing soil led to a significant decline in the measured photosynthetic activity traits expressed as photosynthetic rate (A), stomatal conductance (Gs) and evaporation rate (E) (Table 3). The lowest A and Gs were observed at the highest Ni concentration. In contrast, applying *Azolla* recovered the photosynthetic pigment content and photosynthetic rate (Table 3). Maximal recovery of chlorophyll content and photosynthetic activity was observed at 190 ppm Ni (Fig. 5), where these values exceed one hundred.

**Table 3:** Effect of different concentrations of Ni ion (0, 24, 70, 140, 190 ppm) before and after *Azolla piñnata* treatment on chlorophyll a, chlorophyll b, total chlorophyll content (mg g^−1^ F wt), A (photosynthetic rate) [µmol m^−2^ s^−1^], Gs (stomatal conductance) [mmol m^−2^ s^−1^], E (transpiration rate) [mmol m^−2^ s^−1^], of maize leaves after 30 days from cultivation.

Ni ion conc (ppm)	Treatment	Chlorophyll a	Chlorophyll b	Total Chlorophyll	A	Gs	E
0	Before *Azolla* treatment	6.672 ± 0.29^c^	2.850 ± 0.050^a^	9.522	13.0 ± 1.75^ab^	69.78 ± 10.5^ab^	1.224 ± 0.10^a^
24		5.320 ± 0.15^d^	2.446 ± 0.100^b^	7.766	9.30 ± 0.84^d^	55.054 ± 3.30^c^	0.929 ± 0.04^b^
70		4.616 ± 0.076^d^	1.953 ± 0.046^cd^	6.572	7.38 ± 0.64^e^	45.999 ± 3.7^d^	0.664 ± 0.12^cd^
140		4.253 ± 0.094^d^	1.623 ± 0.023^e^	5.876	5.77 ± 0.64^ef^	30.205 ± 1.40^e^	0.379 ± 0.06^ef^
190		3.570 ± 0.300^e^	0.596 ± 0.202^f^	4.166	4.81 ± 1.95^f^	19.151 ± 4.0f	0.237 ± 0.01^f^
0	After *Azolla* treatment	8.530 ± 0.300^ab^	3.076 ± 0.057^a^	11.606	14.22 ± 1.13^a^	75.973 ± 2.7^a^	1.321 ± 0.14^a^
24		8.140 ± 0.050^a^	2.566 ± 0.030^b^	10.706	12.19 ± 0.85^bc^	62.636 ± 2.9^b^	1.246 ± 0.03^a^
70		7.736 ± 0.115^b^	2.130 ± 0.10^cde^	9.866	11.33 ± 0.33^bc^	54.317 ± 3.0^c^	0.939 ± 0.03^b^
140		6.650 ± 0.105^a^	1.993 ± 0.011^c^	8.643	11.75 ± 0.48^bc^	44.790 ± 2.0^d^	0.729 ± 0.06^bc^
190		5.650 ± 0.173^d^	1.736 ± 0.057^de^	7.386	10.59 ± 1.28^cd^	39.790 ± 2.9^d^	0.491 ± 0.14^de^

Means with the same letters are not significant according to Tukey test at 0.05. Each means value followed by ± standard deviation.

### Oxidative stress (H_2_O_2_ and MDA content)

H_2_O_2_ and MDA have accumulated in maize leaves under Ni-induced stress, resulting in membrane dysfunction and increasing ion leakage (Table 4). Lipid peroxidation, as measured by MDA content, peaked at 190 ppm of Ni, indicating the hazards of high H_2_O_2_ levels. Proline was also accumulated in maize leaves as a protective compound upon the increase in Ni concentration (Table 4). Proline was increased threefold in maize leaves in response to the high Ni concentration (190 ppm) compared to the control.

**Table 4:** Effect of different concentrations of Ni ion (0, 24, 70, 140, 190 ppm) before and after *Azolla piñnata* on H_2_O_2_ [mM H_2_O_2_ g^−1^ F wt], lipid peroxidation [µMDA g^−1^ F wt] content, electrolyte leakage [%] and proline [mM g^−1^ F wt] content of maize leaves after 30 days from cultivation.

Ni ion conc (ppm)	Treatment	H_2_O_2_ content	Lipid peroxidation	Ion leakage	Proline
0	Before* Azolla* treatment	0.157 ± 0.0005^ef^	2.253 ± 0.171^de^	49.788 ± 0.945^fg^	0.82 ± 0.01^fg^
24		0.186 ± 0.0025^d^	2.522 ± 0.045^d^	55.531 ± 1.217^de^	0.94 ± 0.01^f^
70		0.194 ± 0.0050^d^	3.160 ± 0.114^c^	58.133 ± 0.798^cd^	1.30 ± 0.07^d^
140		0.230 ± 0.0070^c^	3.677 ± 0.319^ab^	60.684 ± 0.902^c^	2.11 ± 0.11^b^
190		0.387 ± 0.0130^a^	3.913 ± 0.188^a^	74.456 ± 1.776^a^	2.89 ± 0.12^a^
0	After *Azolla* treatment	0.139 ± 0.0210^ef^	2.063 ± 0.040^e^	49.068 ± 1.100^g^	0.08 ± 0.01^h^
24		0.145 ± 0.0005^f^	2.438 ± 0.045^de^	53.199 ± 0.550^ef^	0.69 ± 0.005^g^
70		0.178 ± 0.0051^de^	2.574 ± 0.041^d^	56.435 ± 0.283^de^	0.80 ± 0.11f^g^
140		0.213 ± 0.0041^d^	2.564 ± 0.070^d^	57.424 ± 0.348^cd^	1.12 ± 0.05^e^
190		0.277 ± 0.0137^b^	3.406 ± 0.011^bc^	66.280 ± 2.866^b^	1.54 ± 0.02^c^

Means with the same letters are not significant according to Tukey test at 0.05. Each mean value followed by ± standard deviation.

The application of *Azolla*-treated Ni solutions reduced the accumulation of H_2_O_2_ and its destructive effect on the plasma membrane by lipid peroxidation (LPOX) in maize leaves. The proline accumulation was retained by *Azolla*-treated Ni solutions to become 1.54 mM g^−1^ F Wt, compared to 2.89 at 190 ppm of Ni.

Increasing the Ni concentration in the soil was accompanied by increasing the maize leaf content of antioxidant compounds (phenolics, flavonoids and total antioxidant capacity) and antioxidant enzyme activity (catalase (CAT) and peroxidases (POX)) (Table 5).

**Table 5:** Effect of different concentrations of Ni ion (0, 24, 70, 140, 190 ppm) before and after *Azolla piñnata* on antioxidants compounds (phenolics, flavonoids [mg g^−1^ D wt] and total antioxidant capacity [mg asc acid g^−1^ F wt] and antioxidant enzymes activity (POX and CAT [mg min^−1^ g^−1^ F wt]) of maize leaves after 30 days from cultivation.

Ni ion conc (ppm)	Treatment	Phenolics	Flavonoids	Total antioxidant capacity	POX	CAT
0	Before *Azolla* treatment	42.91 ± 0.605^f^	10.35 ± 0.05^f^	0.464 ± 0.075^f^	3.02 ± 0.05^e^	11.03 ± 0.09^ef^
24		45.72 ± 0.390^e^	12.95 ± 0.45^e^	0.652 ± 0.006^de^	3.63 ± 0.05^cd^	13.38 ± 1.32^d^
70		49.99 ± 0.330^d^	15.25 ± 0.05^d^	0.767 ± 0.011^c^	3.75 ± 0.11^c^	17.20 ± 0.88^d^
140		55.97 ± 0.745^c^	18.55 ± 0.35^b^	0.873 ± 0.002^b^	4.03 ± 1.45^b^	19.76 ± 0.25^b^
190		68.78 ± 0.425^a^	20.35 ± 0.05^a^	0.971 ± 0.058^a^	4.31 ± 0.25^a^	22.46 ± 0.47^a^
0	After *Azolla* treatment	40.01 ± 0.080^g^	9.400 ± 0.10^g^	0.408 ± 0.016^f^	2.83 ± 0.05^f^	9.360 ± 0.20^f^
24		42.88 ± 0.375^f^	10.75 ± 0.45^f^	0.581 ± 0.008^e^	3.13 ± 0.11^e^	10.30 ± 0.42^f^
70		47.08 ± 0.830^e^	13.30 ± 0.20^e^	0.637 ± 0.003^e^	3.60 ± 0.20^d^	12.57 ± 0.31^de^
140		50.93 ± 0.385^d^	16.30 ± 0.10^d^	0.728 ± 0.014^d^	4.06 ± 0.10^b^	13.72 ± 0.31^d^
190		61.38 ± 1.055^b^	17.25 ± 0.05^c^	0.793 ± 0.011^bc^	4.20 ± 0.05^a^	18.46 ± 0.37^bc^

Means with the same letters are not significant according to Tukey test at 0.05. Each mean value followed by ± standard deviation.

### Antioxidant defense system (enzymatic and non-enzymatic)

**Fig. 6:**
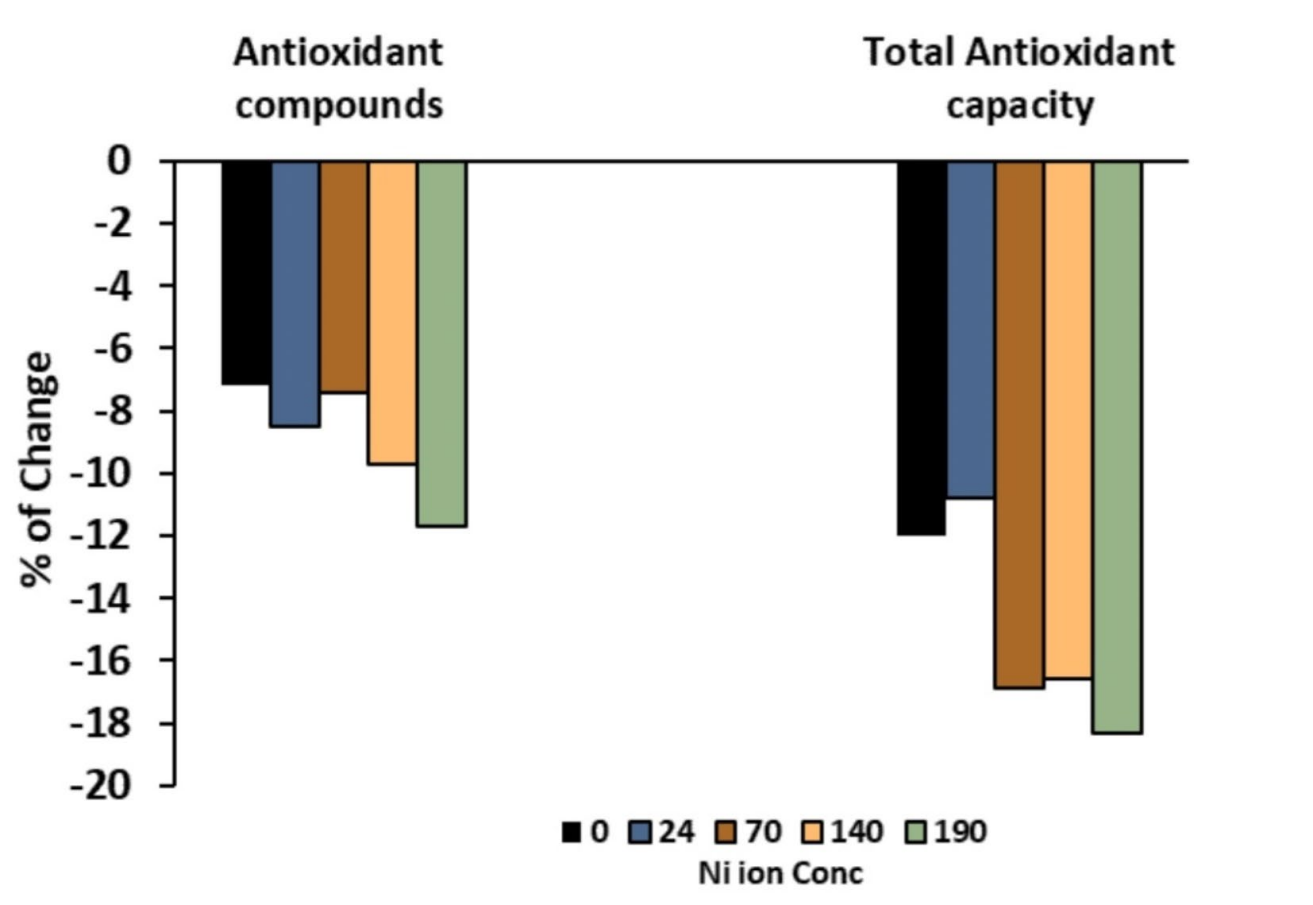
The percentage of change in antioxidants compounds content (phenolics and flavonoids) and total antioxidant capacity in response to *Azolla pinnata* treatment.

Treating Ni solutions with *Azolla* recovered the content of phenolics, flavonoids and antioxidant enzymes activity. The activity of POX and CAT reached 4.20 and 18.46 mg min^−1^g^−1^ F Wt, respectively, compared to 4.31 and 22.46 at untreated 190 ppm of Ni solution (Table 5). The percentage of changes for antioxidant compounds (phenolics, flavonoids) and the total antioxidant capacity revealed negative values, indicating they were positively affected by *Azolla*-treated Ni solutions (Fig. 6).

### Nickel concentration

**Fig. 7:**
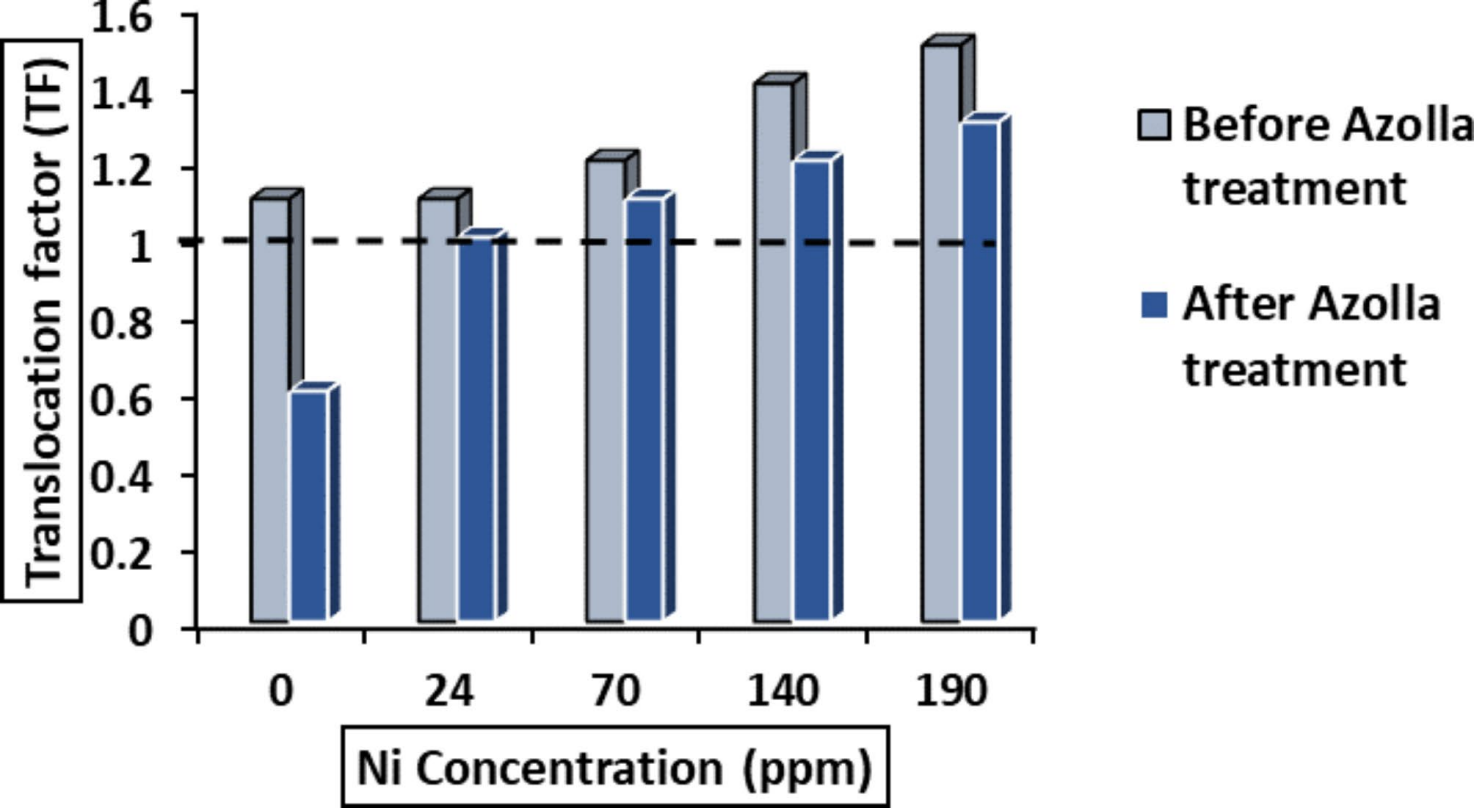
The translocation factor of Ni from root to leavest at different Ni concentration which untreated or treated with *Azolla pinnata.*

In general, Ni concentration and percentage significantly (*p < 0.05*) increased in roots, leaves and grains with increasing Ni concentration in applied solution (Supplementary material S1). The highest concentration of Ni was observed in leaves, while the lowest concentration was in grains. The increment of translocation factor (TF ˃1) of Ni from roots to leaves (Fig. 7) by increasing Ni levels indicates the tendency of Ni to be accumulated in the leaves. Upon treating Ni solution with *Azolla*, the Ni content and percentage in roots, leaves, and seeds were significantly (*p* ≤ 0.05) reduced (Supplementary material S1). The concentration of Ni reached zero in grains at *the Azolla-*treated Ni-24 ppm concentration and the translocation factor of Ni from root to leaf was decreased. The translocation factor was less than or equal to one at low concentrations of Ni (0 and 24 ppm Ni solution) (Fig. 7).

### Yield components

The current results revealed a significant (*p < 0.05*) reduction of the maize yield components with an elevation of Ni concentrations (Table 6). The number and weight of grains per ear, the number of grains per plant, and the weight of 100 grains are the main components of grain yield, which are measured. However, the most affected yield parameter was the weight of 100 grains at 190 ppm Ni, where it verified zero. Zooming on using *Azolla*-treated solutions, all the components of grain yield were enhanced by the application of *Azolla*-treated Ni solutions on maize (Table 6).

**Table 6:** Effect of different concentrations of Ni ion (0, 24, 70, 140, 190 ppm) before and after *Azolla piñnata* treatment on grain yield parameters of maize plant after 6 months from cultivation.

Ni ion conc (ppm)	Treatment	Grain yield			
		Wt of grains ear^−1^	No of grains ear^−1^	Total no of grains plant^−1^	Weight of 100 grains (g)
0	Before* Azolla *treatment	275.1 ± 4.2^c^	97 ± 2.5^a^	1620 ± 5.0^c^	19.3 ± 2.0^b^
24		201.6 ± 1.4^d^	81 ± 1.0^c^	1135 ± 1.5^e^	16.1 ± 0.36^c^
70		123.2 ± 2.1^f^	61 ± 1.5^d^	800 ± 1.1^f^	11.4 ± 0.11^d^
140		16.1 ± 0.76^h^	36 ± 1.0^f^	215 ± 1.5^h^	7.50 ± 0.25^e^
190		2.3 ± 0.321^i^	1.0 ± 0.5^h^	5.0 ± 1.1^j^	00.0 ± 0.00^f^
0	After *Azolla *treatment	347.4 ± 2.4^a^	101 ± 0.5^a^	1900 ± 26.0^a^	23.0 ± 1.00^a^
24		294.1 ± 4.0^b^	86 ± 1.5^b^	1660 ± 5.0^b^	19.8 ± 0.28^b^
70		179.7 ± 7.4^e^	77 ± 2.0^c^	1264 ± 14.0^d^	18.3 ± 0.57^bc^
140		31.6 ± 1.1^g^	43 ± 1.5^e^	316 ± 3.6^g^	16.0 ± 1.00^c^
190		5.6 ± 0.32^i^	20 ± 0.5^g^	60 ± 5.0^i^	6.60 ± 1.03^e^

Means with the same letters are not significant according to Tukey test at 0.05. Each mean value followed by ± standard deviation.

**Fig. 8:**
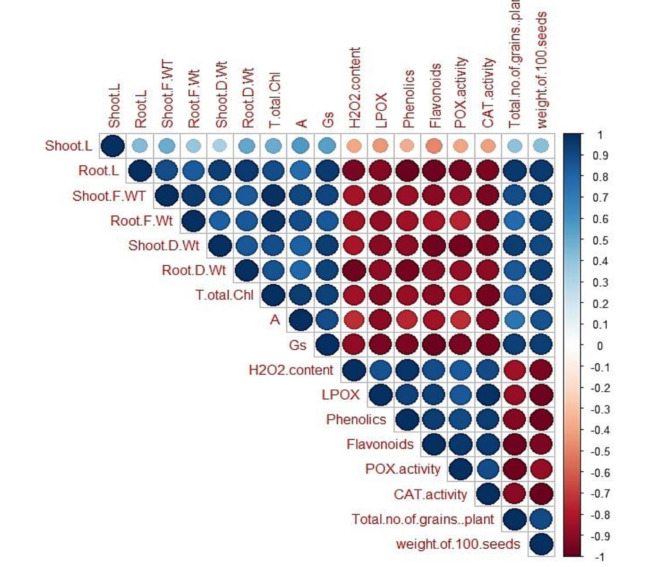
The correlation between all measured paramters (growth parameters, total Chl a, A, Gs, H_**2**_O_2_, LPOX, antioxidant compondsand enzymes, total number of grains per plant and weight of 100 seeds) at different concentrations of Ni ions befor and after *Azolla pinnata* treament.

It was found to be a positive relationship between growth parameters, total Chl, photosynthetic efficiency and yield (Fig. 8). However, those previous parameters were negatively correlated with H_2_O_2_ content and its destructive consequences (lipid peroxidation and electrolyte leakage). In addition, the antioxidant defense component was also negatively correlated with growth factors (particularly after *Azolla* treatment).

## Discussion

The removal or reduction of contaminants (organic or inorganic) has a great interest globally; focusing on the protection of agricultural land^43^. Maize productivity loss mainly affected by the accumulation of heavy metals in soil^44^. Phyto-remediation is an economically and environmentally valuable application to address heavy metal contamination in soil by using plants to remove toxins. Macrophytes are more applicable for this purpose because of their quicker growth and greater biomass productivity^45^. Because of their capacity to hyperaccumulate heavy metals, they may be a confident phytoremediators for metal contaminated industrial and sewage waste water.

The incubated *Azolla* in Ni solutions was damaged showing an abnormal cell wall and disruption of organelles after five days. The same results were observed by Benaroya et al.^46^, where lead (Pb) precipitates were observed in the vacuoles of *A. filiculoides* mesophyll cells as black, dense deposits under a light and transmission electron microscope in the cells of fronds treated with Pb. This might be explained by the existence of specialized ligands that sequester and chelate metal ions in *Azolla* cells^47^. Plants have well-defined heavy metal-binding ligands known as phytochelatins (PCs) and metallothioneins (MTs), according to Joshi et al.^48^. Consequently those chelators, PCs, chelate tightly with metal ions forming complexes and collect them in vacuoles. That is why the heavy metal application greatly elevated MT2 and PCS1 gene expression patterns in *Azolla*, indicating their involvement in metal-remediation ability in a polluted area^49^.

Arif et al.^50^ demonstrated that heavy metals are, firstly, adsorbed in a cationic form with the negative cell wall of *Azolla* cells due to the presence of cellulose, pectins, and other ion exchangers. Secondly, they adsorbed on the cell wall. Finally, they accumulate in the cell^51^.

The reduction in seed germination percentage because of increasing the Ni ions in the surrounding medium could be attributed to its effect on the embryo viability, since this reduction was accompanied with a reduction in the activity of amylases and proteases that hydrolyze the complex stored food (starch and protein) to simple soluble compounds (sugars and amino acids), which supply the embryo axis for growth. Heavy metal ions, such as Ni may be inhibitors for the hydrolytic enzyme activity. In the same way, Zhi et al.^52^ demonstrated the reduction of germination under Ni stress.

The increment in activity of the antioxidant enzymes, peroxidase (POX) and catalase (CAT) at all Ni concentrations indicates an increase in cellular oxidative stress. Raising their activities neutralizes and counters the detrimental effects of reactive species. Similarly, Sethy and Ghosh^53^ found that the high levels of heavy metals in the germinating medium lead to disruption of cellular homeostasis, triggering oxidative stress including alterations in enzymes of the antioxidant defense system. Plant cells are equipped with enzymatic mechanisms to eliminate or reduce ROS-damaging effects^54^.

The activity of the hydrolytic enzymes was recovered by removing Ni ions from the surrounding medium by *Azolla* treatment, increasing the embryo supply with soluble sugars and amino acids necessary for its growth, and increased the germination percentage of maize seeds. Ahmeda and El-Mahdya^55^ mentioned that boosting of amylase activity enhances the mobilization of reserves from seed storage as endosperms or cotyledons for partitioning into embryo. Moreover, *Azolla* treatment reduced the increased activity of POX and CAT in the germinating seeds at all Ni concentrations, indicating a reduction in ROS production. Feng et al.^56^ reported that regulating antioxidant enzyme activities in plants leads to cellular redox balance and prevents cell damage.

Nickel level increasing in the soil considered toxic for maize because it uptakes and accumulates Ni in roots, leaves and grains. Thereby significant decline of the growth of maize was observed especially at higher concentrations of Ni. Nickel toxic effects including the excessive release of reactive oxygen species (ROS) cause a significant reduction in maize growth and functioning. The oxidative stress may lead to a decline in photosynthetic pigments (chlorophyll a and b) and activity in Ni-treated leaves by impacting chlorophyll synthesis enzymes. Similarly, Kumar et al.^57^ indicated that the decrease in the content of chlorophyll is a consequence of Ni toxicity. Magnesium in Chl may be substituted by Ni, which destroys Chl and thylakoid membranes in cabbage leaves and wheat shoots, respectively^58^. Ni reduced Rubisco synthesis and activity, reduced transport electrons to PSII^59^, and restricted Calvin cycle, resulting in a low photosynthesis rate, affecting agricultural plant growth and economics. Excessive Ni exposure may result in non-specific photosynthetic limitations in plants, either directly or indirectly^60^. Ni inhibits electron transport from pheophytin to plastoquinone QA and Fe to plastoquinone QB by altering the structure of carriers such plastoquinone QB and reaction center proteins. Ni ions reduced cytochromes b6f and b559, ferredoxin, and plastocyanin levels in thylakoids, resulting in lower electron transport efficiency^61^. Heavy metal stress causes the production of methylglyoxal (MG), which is a highly reactive cytotoxic alpha-oxoaldehyde compound that interferes with the plant’s normal metabolic activity^62^.

Increasing Ni concentration in soil led to increasing H_2_O_2_ and MDA in maize leaves, resulting in membrane dysfunction, increasing ion leakage because of lipid peroxidation. The Ni atom enhances H_2_O_2_ production by converting solvents into proton sources. Nickel (Ni^3+^) enrich facilitates the reaction intermediates *O, *OH, and *OOH, as well as the transfer of electrons throughout the reaction, enabling the production of hydrogen peroxide^63^. Nevertheless, when Ni and Co united with Fe^2+^, Fe^2+−^H_2_O_2_-mediated lipid peroxidation is encouraged in the occurrence of Ni^2+^ and is repressed in the occurrence of Co^2+^^64^. Lipid peroxidation, expressed by the MDA content, reflects the hazardous effect of the high levels of H_2_O_2_. High MDA (malondialdhyde) content results from the oxidation of the cellular membrane components by nickel, resulting in a high level of lipid peroxidation. This indicated membrane disorganization, so its leakiness has increased, followed by metabolic disturbance. Many authors proved the same outcome in their experiments, such as Rizwan et al.^65^ and Altaf et al.^66^ on rice. Comparable with other metals, Ni has a higher degree of lipid peroxidation due to its higher mobility in plants^67^. MDA accumulation and its negative influence on plant biomass and nutritional balance are attributed to ROS overproduction, which induces oxidative stress. This effect is considered a common response in maize plants to Cd^68^ and Ni stress. By the same way, Kumar et al.^69^ showed the raising of H_2_O_2_ and MDA under Cr stress in *H. annuus* L.

Proline has accumulated threefold in maize leaves, as a protective compound, upon increasing Ni concentration to 190 ppm. Osmotic adjustment metabolites (especially proline) and the antioxidant system have a significant role in cell protection against metal toxicity. Osmotic adjustment metabolites help to maintain the turgor pressure of the cell and are considered a metal chelator according to Bashir et al.^70^. They are involved in protein stabilization, direct scavenging of ROS, intracellular redox homeostasis balance (e.g., NADP^**+**^/NADPH and GSH/GSSG ratios), and cellular signaling pathways^71^. Increasing the Ni concentration in the soil was accompanied by increasing the maize leaf content of phenolics, flavonoids and total antioxidant capacity, as well as the antioxidant enzymes POX and CAT, to prevent the oxidative stress. Metal ions breakdown lipid hydroperoxide (LOOH) by hemolytic breakage of the O–O link, producing lipid alkoxyl radicals that trigger free radical reactions. Phenolic antioxidants prevent lipid peroxidation by trapping the lipid alkoxyl radical. The activity of molecules is determined by their structure, as well as the amount and location of their hydroxyl group^72^.

Maize plants exposed to Ni stress significantly buildup phenolics, phytoalexins, and activated antioxidant enzymes such as CAT and POXs, as well as enzymes controlling phenylpropanoid and isoflavonoid biosynthesis, which are thought to be important regulators of stress tolerance^73^. Along with the accumulation of H_2_O_2_, phenolics, especially flavonoids, act as H_2_O_2_ scavengers^74^. Flavonoids create stable complexes with heavy metal ions, preventing oxidative stress from developing^75^. CAT may help in the defense against H_2_O_2_ accumulation, which was previously reported in the current study. This result was in harmony with the results obtained by^67^ on maize. The enhancement of antioxidant enzyme activities has a crucial role in overcoming Ni stress, and Ni treatments significantly influence enzymatic activities^76^. It was suggested that antioxidant enzymes (SOD, POD, CAT, and APX) activity is higher in tolerant species of *Solanum lycopersicum* L. (under Pb stress) than those sensitive, indicating their vital role under metal stress^77^.

To provide safe and nutritious agricultural products, sustainable agriculture is essential. Heavy metal contaminated agriculture lands have a deleterious impact on crop growth, development, and production, indicating a challenge for sustained agriculture^78^. So, the mitigation of the Ni-affected soil and the prevention of Ni from entering agricultural environments are critical to overcoming this challenge. This issue recommends a variety of remediation methods to recover the heavy metal-contaminated soil. Many studies deal with the Ni remediation by *Jatropha curcas* and *Pongamia pinnata*^79^, while others used biochar and ryegrass^80^. In the current study, we used *Azolla* for Ni remediation from soil to restore the environmental quality. *Azolla* has significant features that make it a better plant system for phytoremediation than many other macrophytes. In the present work, *Azolla* treated Ni-contaminated irrigation water protected maize leaves by decreasing ROS (H_2_O_2_) generation and re-establishing membrane configuration and photosystem activity by increasing chlorophyll content and carbon dioxide assimilation. Thus, plants respond biochemically by reducing oxidative stress and inflammation, improving growth metrics. This result was supported by Shedeed and Farahat^81^ study on the beneficial role of *Azolla* under Co and Cd stress.

Focusing on the percentage of changes in the measured parameters (during germination and vegetative growth), the calculated data emphasizes the impact of the different treatments, indicating the crucial role of *Azolla* in mitigating the toxicity of Ni on maize plants during germination and vegetative growth. *Azolla*, a free-floating, fast-growing, nitrogen-fixing pteridophyte, appears to be an outstanding option for heavy metal removal, disposal, and recovery from damaged aquatic environments^82^.

The tendency of Ni to accumulate in the maize leaves is more than the root was also observed in the Amjad et al.^67^ study, where they found that the translocation factor of Ni from root to leaf increased at a Ni concentration of 40 mg l^−1^ in maize hybrid Pioneer. The accumulation of Ni ions in the maize leaves indicates the impact of this heavy metal on metabolic activities. Amjad et al.^67^ confirmed an increase in Ni content in shoots and roots by increasing Ni concentration in maize. Ni is absorbed by roots via the iron (Fe) absorption transporter Iron-regulated transporter 1 (IRT1) and subsequently translocated (translocation factor ˃ 1) to shoots via the xylem^83^. IREG2 knockdown reduces root Ni while increasing shoot Ni, indicating that IREG2 may influence the efficiency of Ni translocation from roots to aerial parts^84^.

The reduction in maize yield with elevation of Ni concentrations could be attributed to the nutrient imbalance, because of the reduction of photosynthesis, as well as the stomatal conductance and evaporation rate that affect water and nutrient absorption by roots. Yield loss poses a substantial hazard to global food safety, hence it is critical to maximize crop yield potential in both normal and stressed situations. Nickel induced crop yield reduction due to the disturbance of nutrient absorption by roots in the presence of Ni^85^. Reduced yields under Ni stress have been recorded in mungbean^86^ and *Hibiscus sabdariffa*^87^. Photosynthesis in heavy metal-stressed plants decreased and the nitrogen content in the roots and leaves decreased in mungbean and chickpea^88^. So, this nutrient imbalance negatively affects the grain development in maize. *Azolla-*treated Ni solutions increased CO_2_ fixation compared to the untreated Ni solutions so the grain yield parameters would be improved. Recent research has shown compelling evidence that boosting photosynthetic efficiency via various systems may compromise a route to enhance agricultural production potential. Photosynthesis drives many biological activities such as crop production^89^.

## Conclusion

Although plants need nickel as a crucial element for ideal growth, excess Ni in growing medium are harmed maize by the high disturbance range of physiological activities such as germination, growth, photosynthesis, enzymatic activities and yield, especially at high concentrations of Ni ions [190 ppm]. Nickel induced the lipid oxidation by releasing high rates of H_2_O_2_, destabilizing membranes in the cell and chloroplast. That influences the structural and functional integrity of chloroplast membrane, affecting electron transport chain and carbon fixation. Non-enzymatic (phenolics and flavonoids) and enzymatic (POX and CAT) antioxidant systems functioned as efficient scavenging system, preventing distribution of ROS to prevent further damage in the cell under Ni stress. Ni accumulated in leaves in higher concentration, showing translocation factor more than unity (TF ˃ 1) for root-leaf. *Azolla* treatment recovered the metabolic disruption in maize caused by Ni toxicity. *Azolla*-treated Ni-solutions enhanced growth parameters, photosynthesis and yield. Thus, *Azolla* is cheap and applicable strategy for purifying heavy metal-contaminated water. Thus, one of our future prospects to use *Azolla* with mixed heavy metal polluted irrigation water and give a complete picture of translocation, partitioning and toxicity of different heavy metals in plants.

## Electronic supplementary material

Below is the link to the electronic supplementary material.

Supplementary Material 1

## Data Availability

Data is provided within a supplementary information file besides the main manuscript file.
