# Supplementary material for: Azolla pinnata as a phytoremediator: improves germination, growth and yield of maize irrigated with Ni-polluted water
Source: Sci Rep. 2024 Sep 27;14:22284. doi: 10.1038/s41598-024-72651-1 (PMC11437153; doi:10.1038/s41598-024-72651-1)
Supplement: Supplementary file 1 — Supplementary Material 1 [file 41598_2024_72651_MOESM1_ESM.docx]

**S. Effect of different concentrations of Ni ion (0, 24, 70, 140, 190 ppm) before and after *Azolla piñnata* treatment on antioxidant enzymes activity (CAT and POX) [**mg g^-1^ F Wt min^-1^] **of maize seeds (7-d-old).**

| **Ni ion conc**  **(ppm)** | **Treatment** | **POX** | **CAT** |
| --- | --- | --- | --- |
| **0** | ***Before Azolla treatment*** | 18.07±1.57^ef^ | 0.272±0.002^g^  0.422±0.008^d^  0.547±0.033^c^ |
| **24** |  | 35.00±2.40^d^ |  |
| **70** |  | 48.75±0.82^c^ |  |
| **140** |  | 62.75±2.75^b^ | 0.650±0.011^b^ |
| **190** |  | 74.25±0.25^a^ | 0.802±0.000^a^ |
| **0** | ***After***  ***Azolla treatment*** | 16.65±0.01^f^ | 0.287±0.007^fg^ |
| **24** |  | 23.10±2.30^e^ | 0.329±0.003^fg^ |
| **70** |  | 31.42±3.55^d^ | 0.331±0.005^fg^ |
| **140** |  | 46.18±1.68^c^ | 0.340±0.010e^f^ |
| **190** |  | 58.63±0.65^b^ | 0.397±0.053d^e^ |

Means with the same letters are not significant according to Tukey test at 0.05. Each mean value followed by ± standard deviation.

**S1. Effect of different concentration of Ni ions (0, 24, 70, 140, 190 ppm) before and after *Azolla piñnata* treatment on Ni concentration** [µg kg^-1^ D Wt] **in roots, leaves and seeds of maize after 30 days from cultivation**

| **Ni ion conc**  **(ppm)** | **Treatment** | **Ni concentration** | | |
| --- | --- | --- | --- | --- |
|  |  | **Roots** | **leaves** | **Seeds** |
| **0** | ***Before Azolla treatment*** | 11.5±0.50^g^ | 13.0±0.50^h^ | 0.0±0.00^g^ |
| **24** |  | 43.1±3.00^e^ | 46.0±0.17^f^ | 3.1±0.01^d^ |
| **70** |  | 50.4±0.45^d^ | 60.3±0.55^e^ | 4.1±0.24^c^ |
| **140** |  | 70.0±0.10^c^ | 95.0±0.00^c^ | 5.0±0.20^b^ |
| **190** |  | 98.0±0.05^a^ | 150.0±0.10^a^ | 8.0±0.03^a^ |
| **0** | ***After Azolla treatment*** | 9.30±0.49^g^ | 6.00±0.57^i^ | 0.0±0.00^g^ |
| **24** |  | 33.1±0.28^f^ | 35.0±0.05^g^ | 0.0±0.00^g^ |
| **70** |  | 41.0±0.10^f^ | 46.1±0.10^f^ | 0.51±0.01^f^ |
| **140** |  | 68.1±0.51^c^ | 85.0±0.05^d^ | 1.50±0.10^e^ |
| **190** |  | 80.1±0.05^b^ | 103±0.00^b^ | 5.0±0.10^b^ |

Means with the same letters are not significant according to Tukey test at 0.05. Each mean value followed by ± standard deviation.
